# Natural products modulate social behavior and gut microbiota in a valproic acid-induced zebrafish model of autism spectrum disorder

**DOI:** 10.17305/bb.2026.14302

**Published:** 2026-06-26

**Authors:** Oana-Georgiana Oprea, Ioana-Miruna Balmus, Tudor Ilie, Gabriel Plavan, Mircea Nicusor Nicoara, Lucian Gorgan, Anca Trifan, Bogdan Stoica, Bogdan-Ionel Tamba, Alin Ciobica, Mitica Ciorpac

**Affiliations:** 1 Doctoral School of Biology, Faculty of Biology, Alexandru Ioan Cuza University of Iasi, Iasi, Romania; 2 Department of Exact Sciences and Natural Sciences, Institute of Interdisciplinary Research, Alexandru Ioan Cuza University of Iasi, Iasi, Romania; 3 CENEMED Platform for Interdisciplinary Research, “Grigore T. Popa” University of Medicine and Pharmacy, Iasi, Romania; 4 Synergy Plant Products, Prejmer, Brasov, Romania; 5 Department of Biology, Faculty of Biology, Alexandru Ioan Cuza University of Iasi, Iasi, Romania; 6 Doctoral School of Geosciences, Faculty of Geography and Geology, Alexandru Ioan Cuza University of Iasi, Iasi, Romania; 7 Department of Gastroenterology, Faculty of Medicine, Grigore T. Popa University of Medicine and Pharmacy Iasi, Iasi, Romania; 8 Institute of Gastroenterology and Hepatology, “Sf. Spiridon” County Clinical Emergency Hospital, Iasi, Romania; 9 Discipline of Biochemistry, Faculty of Medicine, Grigore T. Popa University of Medicine and Pharmacy, Iasi, Romania; 10 Advanced Research and Development Center for Experimental Medicine “Prof. Ostin C. Mungiu”—CEMEX, Grigore T. Popa University of Medicine and Pharmacy Iasi, Iasi, Romania; 11 Department of Pharmacology, Clinical Pharmacology and Algesiology, Grigore T. Popa University of Medicine and Pharmacy Iasi, Iasi, Romania; 12 “Ioan Haulica” Institute, Apollonia University, Iasi, Romania; 13 Academy of Romanian Scientists, Bucharest, Romania

**Keywords:** Zebrafish, valproic acid, autism, honey, plants extract, social behaviour, anxiety

## Abstract

Natural products rich in honey- and plant-derived bioactive compounds are increasingly investigated as modulators of the gut–brain–microbiome axis, a pathway implicated in autism spectrum disorder (ASD)-related behavioral and gastrointestinal alterations. This study evaluated whether two novel formulations, a honey-based product (API) and a plant extract-based product (MOD), modulate exploratory, anxiety-like, and social behavior and intestinal microbiota composition in an adult valproic acid (VPA)-induced zebrafish model of ASD. Adult wild-type zebrafish were assigned to control, VPA (300 µM), API (500 mg/L), MOD (500 mg/L), VPA+API, and VPA+MOD groups. Behavioral outcomes were assessed using the novel tank test and a T-maze social behavior test, while intestinal microbiota was profiled by 16S rRNA gene sequencing using an Oxford Nanopore platform. VPA exposure induced mild locomotor and anxiety-like behavioral alterations, whereas API and MOD further modified exploratory and social behavioral readouts in both healthy and VPA-treated zebrafish. Both formulations increased the time spent near the social stimulus; however, this finding should be interpreted cautiously because treatment-associated hypolocomotion and altered exploratory activity may influence T-maze performance. Microbiota profiling revealed a Pseudomonadota-dominated community across all groups, with descriptive treatment-associated shifts in secondary phyla and diversity patterns, particularly in combined VPA+API and VPA+MOD groups. However, no significant differences in alpha diversity, beta diversity, or genus-level abundance remained after multiple-testing correction. These findings suggest that API and MOD may modulate behavioral outcomes in adult zebrafish exposed to VPA, while their effects on gut microbiota remain exploratory and require validation in larger, adequately powered studies.

## Introduction

The core symptoms of autism spectrum disorder (ASD), characterized by impaired social behavior, communication deficits, and repetitive behaviors, are frequently accompanied by numerous comorbidities, such as affective disorders and gastrointestinal impairments. These co-occurring conditions often arise from shared or consequential pathogenic mechanisms [1]. Research has established a correlation between altered gastrointestinal functions and impaired behavior, highlighting the bidirectional communication within the gut-brain-microbiome axis [1, 2]. Recently, there has been growing interest in the therapeutic potential of honey and herbal preparations for various health conditions, including their role in mitigating the progression of neurodevelopmental disorders like ASD [3–7]. Previous studies have reported the capacity of various honey derivatives—including bee honey [3, 8], raw honey [9], Tualang honey [10], polyfloral honey [11], Kelulut honey [12], Manuka honey [13], and honey enriched with additives [14]—to enhance cognitive functions.

The zebrafish (*Danio rerio*) serves as a robust model for investigating neurodevelopmental disorders such as ASD, owing to its significant advantages, high genetic homology, and physiological similarities with humans, particularly concerning neurocognitive functions, the gastrointestinal system, and the microbiome [1, 15–18]. While valproic acid (VPA) is an antiepileptic agent with diverse therapeutic applications in epilepsy, bipolar disorder, and migraine prophylaxis [19–21], its involvement in altering neurodevelopmental mechanisms has been linked to neurocognitive and behavioral deficits characteristic of neurodevelopmental disorders [22, 23]. Consequently, VPA administration in zebrafish models can induce ASD-associated behavioral symptoms, including impaired social behavior, altered exploratory and locomotor activity, and manifestations of aggressive and anxiety-like behaviors [24]. Furthermore, a connection between neurocognitive impairments and gastrointestinal dysbiosis has been observed in zebrafish models treated with ASD-related chemical compounds (e.g., drugs, pesticides, pollutants, heavy metals) [25–29].

In this context, our study aimed to evaluate the potential of two novel formulations, one based on honey and the other on plant extracts, to modulate locomotor and socio-affective behaviors in an adult zebrafish model of ASD induced by VPA. Additionally, we sought to assess VPA’s capacity to induce gut dysbiosis, explore the potential correlation between VPA-associated neurocognitive impairments and gut dysbiosis, and determine if our natural formulations could ameliorate neurocognitive symptoms and promote gut microbiota homeostasis.

**Figure 1.**
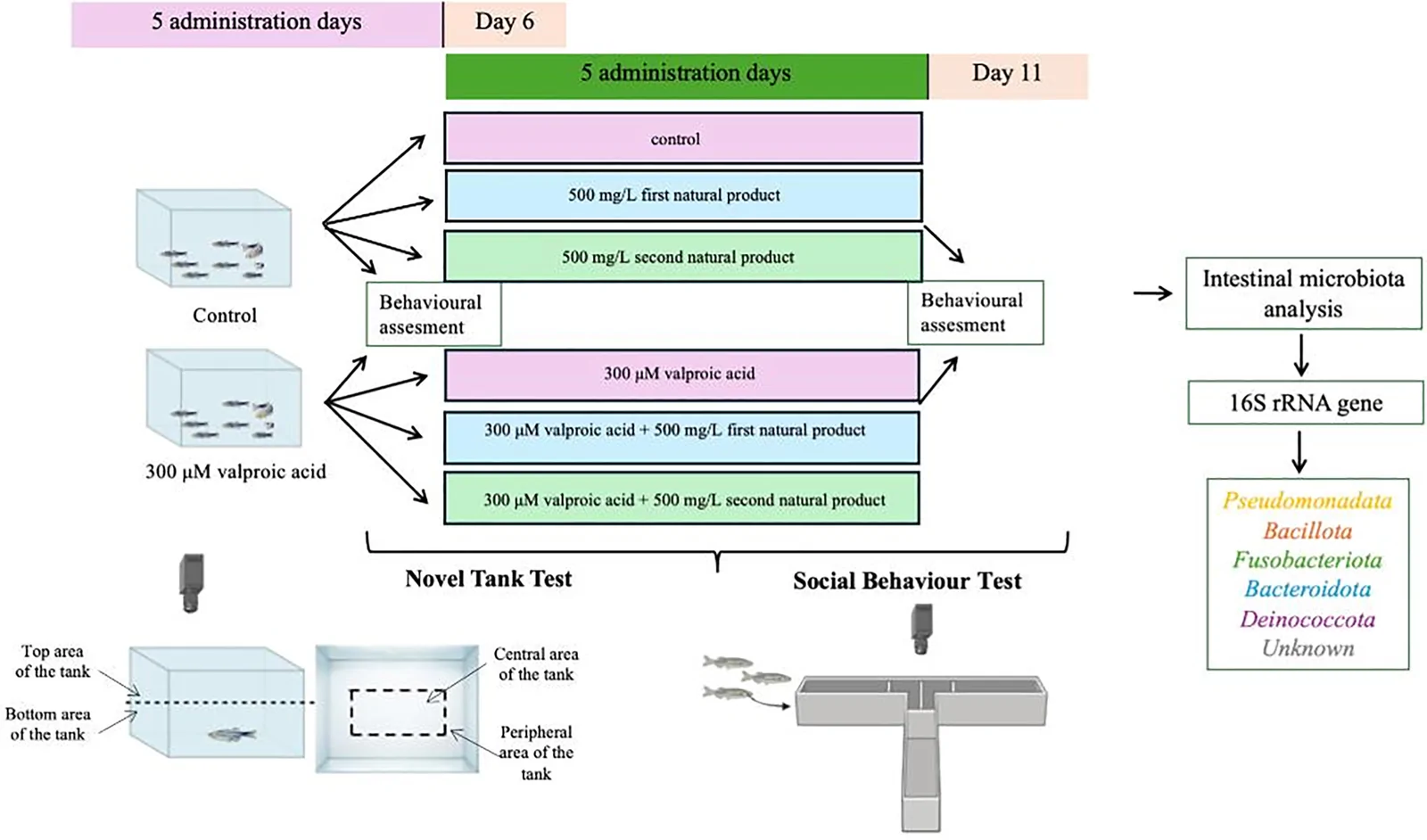
**Experimental design of the valproic acid-induced adult zebrafish model and natural product treatments.** Adult wild-type zebrafish were initially assigned to control and VPA-exposed groups, with VPA administered at 300 µM for 5 days to induce ASD-like behavioral alterations. After the initial exposure period, fish were allocated into six experimental groups: CTR, VPA, API, MOD, VPA+API, and VPA+MOD. API and MOD were administered at 500 mg/L for 5 days, either alone or following VPA exposure. Behavioral assessment was performed using the novel tank test to evaluate exploratory and anxiety-like behavior and the social behavior test to assess social interaction in a T-maze paradigm. At the end of the experimental protocol, intestinal samples were collected for exploratory microbiota profiling based on 16S rRNA gene analysis, with taxonomic evaluation focused on major bacterial phyla identified across experimental groups. **Abbreviations**: API, first natural product based on honey; ASD, autism spectrum disorder; CTR, control; MOD, second natural product based on plant extracts; NTT, novel tank test; rRNA, ribosomal RNA; VPA, valproic acid.

## Materials and methods

### Zebrafish accommodation

Thirty wild-type zebrafish (*Danio rerio*), aged 2–3 months, with an equal sex ratio (1:1), were obtained from an authorized local breeder. Fish were housed for two weeks in a 50 L tank under standard conditions: water temperature 27 ± 1^∘^C, pH 7.5, dissolved oxygen 7.20 mg/L. Daily water changes were performed, and a 14:10 h light/dark cycle was maintained. Fish were fed daily with standard fish food (TetraMin flakes, Germany) according to recommended feeding ratios [30].

### Experimental design

Following the accommodation period, two initial experimental groups were established in distinct tanks under standard laboratory conditions, each containing 15 fish (*n* ═ 15 fish/group): Control (CTR) and VPA (VPA – 300 µM). These groups received their respective treatments for five days. Subsequently, the initial groups were further divided into six experimental groups (*n* ═ 5/group) as depicted in Figure 1: CTR, VPA (300 µM VPA), honey-based product (API) (500 mg/L of the first natural product based on honey), MOD (500 mg/L of the second natural product based on plant extracts), VPA + API (300 µM VPA and 500 mg/L of the first natural product), and VPA + MOD (300 µM VPA and 500 mg/L of the second natural product). The VPA treatment was discontinued after the initial five days, but the VPA group remained untreated until the study’s conclusion.

VPA (Sigma Aldrich, Germany) and the first and second natural products (received directly from the producer) were administered continuously in the housing tank water (final volume 5 L) for five days. Solutions were prepared daily, and water was completely changed to prevent the accumulation of residual compounds. Treatments were performed in a blind manner by one investigator.

The first natural product, API, is a mixture consisting of polyfloral honey (90.39%) enriched with crude royal jelly (7%) and plant extracts. The plant extracts include *Spirulina arthrospira* (0.2% hydrosaline extract containing 25% phycocyanin), *Arctium lappa* L. (1% aqueous extract), *Matricaria chamomilla* (1% aqueous extract and 0.06% essential oil), *Eleutherococcus senticosus* (0.1% root extract containing 0.8% eleutherosides), *Silybum marianum* (0.1% hydroalcoholic fruit extract containing 80% silymarin)*, Panax ginseng (0.05% dried hydroalcoholic root* extract, 4:1 maltodextrin, containing 4% ginsenosides), *Astragalus membranaceus* (0.05% 20:1 hydroalcoholic root extract containing 50% polysaccharides), *Boswellia serrata* (0.01% micellar emulsified extract containing 12% boswellic acid)*, Salvia sclarea (0.02% essential oil)*, and *Salvia officinalis (0.02% essential oil).* Doses and administration methodology were based on previous work by Păduraru et al. [14].

The second natural product, MOD, was administered at similar doses and consists of a mixture of plant extracts: *Betula alba* (11.15% stem sap-originating xylitol), aqueous extracts of *Rubus idaeus* (29.25% 20:1 leaf extract), *Vaccinium vitis-idaea* (28.25% 20:1 leaf extract), *Vaccinium myrtillus* (15.45% 20:1 leaf extract), *Cichorium intybus* (10.5% root extract containing 97% oligofructose), *Saccharomyces cerevisiae* (0.75% extract containing 60% nucleosides), *Hericium erinaceus* (0.75% fruit body extract containing 30% polysaccharides), *Tremella fuciformis* (0.75% 8–10:1 fruit body extract containing 30% polysaccharides), *Pleurotus ostreatus* (0.75% 11-14:1 fruit body extract containing 30% polysaccharides), *Citrus paradisi* (0.6% dried hydroalcoholic seed extract), and essential oils: *Citrus paradisi* (0.6%), *Mentha viridis* (0.6%), and *Citrus sinensis* (0.6%). To avoid potential conflicts of interest, producer brand names were anonymized and encoded as API (LN.2865/06.2025/06.2028) and MOD (LN.6853/04.2025/04.2027).

### Behavioral assessment

Twenty-four hours after the final treatments, behavioral assessments were conducted to evaluate exploratory, social, and anxiety-like behaviors. These assessments were performed by an investigator blinded to the treatment groups.

### Novel tank test (NTT)

The Novel Tank Test (NTT), conducted to evaluate exploratory and anxiety-like behaviors, followed established methodology [31–33]. The test utilized a 6 L rectangular tank, virtually divided into bottom and top areas (from the side view) and peripheral and central areas (from above). After a 30-second acclimatization period, each zebrafish explored the new environment for 4 min. General locomotor parameters recorded for exploratory behavior included total swimming distance (cm), average swimming velocity (cm/s), total inactivity time (s), frequency of clockwise and counter-clockwise rotations (#), and freezing behavior (s). Specific anxiety-like behavior parameters included time spent in the top area (s), frequency of bottom-to-top crossings (#), time spent in the bottom area (s), thigmotaxis (s) (time spent in the peripheral area), and frequency of peripheral-to-central crossings (#). Fish activity was recorded and analyzed using EthoVision XT 16 software (Wageningen, Netherlands).

### Social behavior test

The social behavior test was performed in a T-maze divided into three arms (left, right, and central), in accordance with literature guidelines [34]. A social stimulus, consisting of three conspecifics, was placed at the end of the left arm, separated by a plexiglass wall. After a 30-second acclimatization at the start point, the social behavior test proceeded for 4 min. Specific parameters for social behavior included: time spent near the social stimulus (s) in the left arm, frequency of entries into the social stimulus arm (#), and time spent in the other maze arms (s) (right arm, central arm, and decision point). Additionally, general locomotor performance parameters—total swimming distance (cm), average swimming velocity (cm/s), total inactivity time (s), and frequency of clockwise and counter-clockwise rotations (s)—were assessed to evaluate the zebrafish’s overall locomotor activity. Zebrafish activity was recorded and processed using EthoVision XT 16 software (Wageningen, Netherlands).

### Microbiota analysis

*Sample Collection and Storage.* At predefined experimental endpoints, fish were euthanized in accordance with approved ethical protocols [35, 36]. The microbiota analysis was exploratory. From the 30 fish in the experimental design, three individuals were randomly selected from each experimental group for intestinal microbiota profiling, resulting in 18 biological samples (CTR, VPA, API, VPA + API, MOD, and VPA + MOD; *n* ═ 3/group). Although the sequencing kit supported 24 barcodes, only barcodes 01–18 corresponded to experimental fish and were included in the biological analyses. The entire intestine was aseptically dissected using sterile forceps and scissors, taking care to avoid contamination from surrounding tissues. When necessary, intestinal samples were gently rinsed with sterile phosphate-buffered saline to remove external debris. Each intestine was immediately transferred into a sterile, labeled microcentrifuge tube and snap-frozen without cryoprotectant. Samples were stored at −80^∘^C until DNA extraction. All samples were processed individually to minimize cross-contamination. Extraction blanks or sequencing negative controls were not included; this is acknowledged as a limitation of the exploratory microbiota analysis.

### Microbial genomic DNA extraction

Genomic DNA was extracted from intestinal samples using the Maxwell^®^ RSC Fecal Microbiome DNA Kit (Promega, Madison, WI, USA) on a Maxwell RSC Instrument, following the manufacturer’s instructions. The concentration and purity of microbial DNA were assessed using a SpectraMax QuickDrop ultraviolet–visible (UV–Vis) spectrophotometer (Molecular Devices, San Jose, CA, USA).

### 16S rRNA gene amplification and library preparation

Bacterial community profiling was performed by sequencing the 16S rRNA gene using the Rapid Sequencing DNA–16S Barcoding Kit 24 V14 (SQK-16S114.24, Oxford Nanopore Technologies, Oxford, UK). For each sample, 10 ng of genomic DNA was subjected to barcoded PCR amplification using kit-provided primers. Amplified products were quantified using the Equalbit 1× dsDNA HS Assay Kit (EQ121-01, Vazyme International LLC, Nanjing, China) on a QFX Fluorometer (DeNovix Inc., Wilmington, DE, USA), pooled in equimolar concentrations, and purified using AMPure XP beads. Rapid sequencing adapters were ligated according to the manufacturer’s protocol. Prepared libraries were kept on ice and loaded immediately onto the flow cell.

### Sequencing and bioinformatic analysis

Sequencing was performed on a MinION Mk1B device using an R10.4.1 flow cell (FLO-MIN114, Oxford Nanopore Technologies, Oxford, UK). Raw sequencing data generated in POD5 format were basecalled using Dorado basecaller (v5.2.0; Oxford Nanopore Technologies) with the dna_r10.4.1_e8.2_400bps_hac model. Basecalling was performed using graphics processing unit (GPU) acceleration with Compute Unified Device Architecture (CUDA) support, recursive processing enabled, and the SQK-16S114-24 kit specified. Reads were output in FASTQ format.

Demultiplexed FASTQ files were analyzed using the epi2me-labs/wf-16s Nextflow workflow (v1.6.0) executed with the standard profile. Taxonomic classification was performed using the minimap2 classifier (minimap2 v2.28-r1209) against the National Center for Biotechnology Information (NCBI) 16S/18S reference database. The wf-16s read-length filters were set to min_len = 800 bp and max_len = 2000 bp; other workflow parameters remained at default values unless otherwise specified. Raw genus-level counts for the same barcode were summed across the two sequencing runs before downstream analysis. The final dataset was restricted to barcodes 01-18, corresponding to the 18 biological samples selected for microbiota profiling.

### Ethical statement

All procedures were performed according to the Recommendation of the European Union Commission of 18 June 2007 and the Directive of the European Parliament and of the Council of the European Union of 22 September 2010. The study was approved by the Ethics Committee of the Faculty of Biology of “Alexandru Ioan Cuza” University of Iasi (122/12.05.2025).

### Statistical analysis

GraphPad PRISM software (version 10.0.0 for Windows, Boston, MA, USA) was used for statistical analysis of numerical data. Results were graphically expressed as box-and-whisker plots, where boxes represent the interquartile range, center lines indicate the median, whiskers are ±SD, and asterisks represent significant *P*-values from one-way analysis of variance (ANOVA). One-way ANOVA, followed by post-hoc analyses (Tukey’s honestly significant difference [HSD] test), was performed to test differences between groups. A *P*-value less than 0.05 was considered statistically significant for a given F value. For microbiota analyses, due to substantial variation in sequencing depth among samples, a minimum inclusion threshold of ≥10 genus-level classified reads was applied. Samples below this threshold were excluded from diversity analyses. Alpha-diversity indices were calculated after rarefaction to 100 reads, corresponding to the lowest sequencing depth among samples, using 1000 rarefaction iterations. Alpha-diversity indices were compared among groups using the Kruskal-Wallis test, with Benjamini-Hochberg false discovery rate (FDR) correction applied across indices. Beta diversity was assessed at the genus level using Bray-Curtis dissimilarity calculated from total-sum-scaled relative abundance data. Group separation was tested using permutational multivariate analysis of variance (PERMANOVA) with 9999 permutations. Differential abundance testing was performed at the genus level using the Kruskal-Wallis test on relative abundance data; taxa were retained for testing if present in at least two samples and if their maximum relative abundance was ≥1%. Resulting *P*-values were adjusted using the Benjamini-Hochberg FDR procedure.

## Results

### The effect of API and MOD on locomotion and affective behavior

In the VPA-treated group, impairments in locomotor behavior were observed, characterized by increased total swimming distance, decreased average swimming velocity, and changes in clockwise and counter-clockwise rotation frequency; however, these differences were not statistically significant (Figure 2A–F). Moreover, increased freezing behavior and total inactivity time in the VPA-treated group could indicate anxiolytic states (Figure 2C, D). Similar changes in locomotor behavioral parameters were also observed in the VPA+API group, but no statistically significant effects were registered (Figure 2A–F). Similarly, no statistically significant changes were observed in the API group (Figure 2A–F). Additionally, increased freezing behavior and increased total inactivity time were observed in the VPA+API group, while no major changes were observed when healthy individuals were exposed to API (Figure 2C, D).

**Figure 2.**
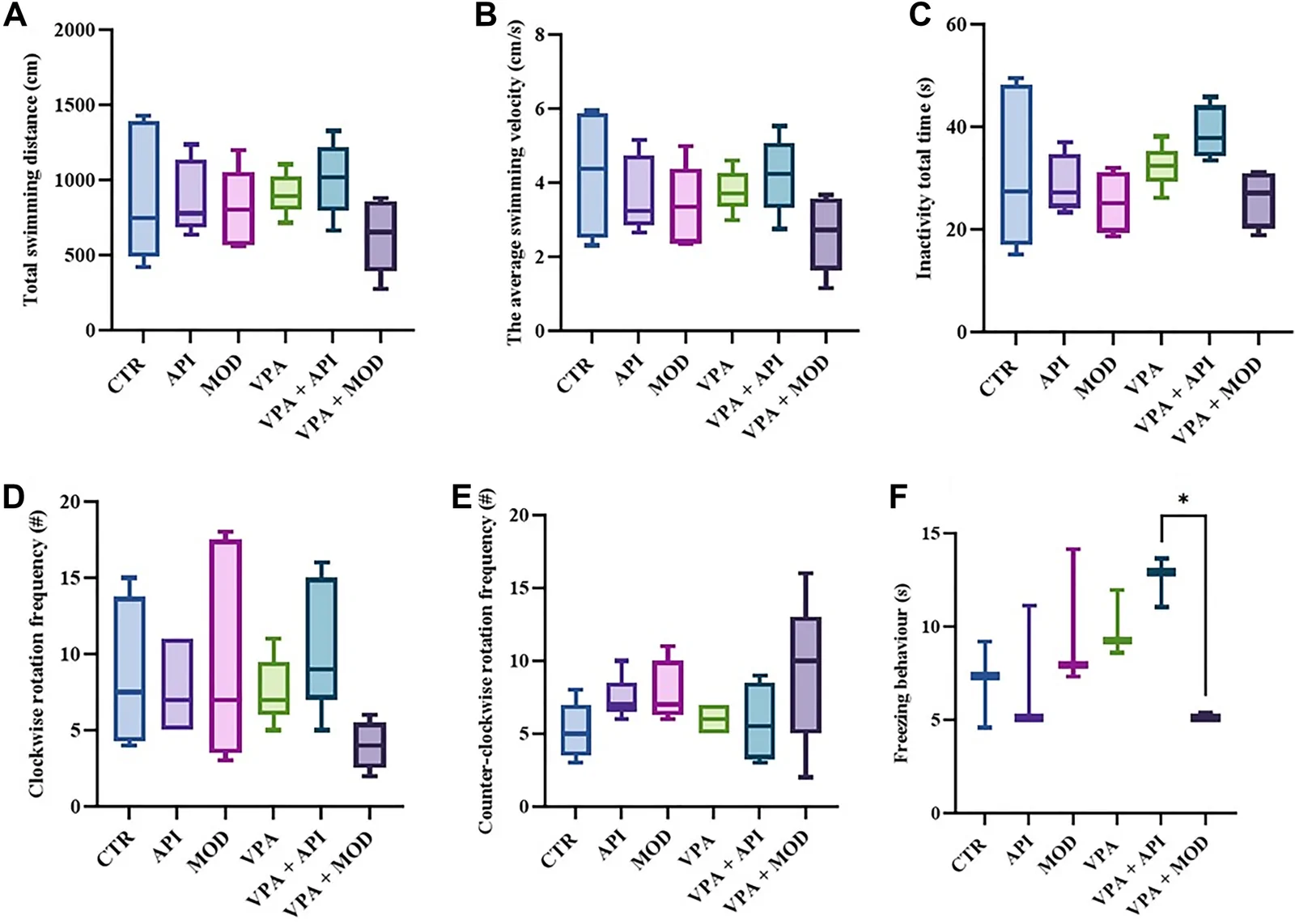
**Effects of API and MOD on locomotor and exploratory behaviour in the novel tank test.** Locomotor performance was assessed 24 h after the final treatment using the novel tank test. The evaluated parameters included: (A) total swimming distance, (B) average swimming velocity, (C) total inactivity time, (D) clockwise rotation frequency, (E) counter-clockwise rotation frequency, and (F) freezing behaviour. VPA exposure was associated with altered locomotor activity and increased inactivity/freezing-related responses, while API and MOD differentially modulated these behavioural parameters in healthy and VPA-treated zebrafish. A significant reduction in freezing behaviour was observed in the VPA+MOD group compared with the VPA+API group. Data are presented as box-and-whisker plots; boxes indicate the interquartile range, horizontal lines indicate the median, and whiskers indicate SD. Statistical significance was assessed using one-way ANOVA followed by post hoc analysis. *P < 0.05. **Abbreviations**: ANOVA, analysis of variance; API, first natural product based on honey; CTR, control; MOD, second natural product based on plant extracts; NTT, novel tank test; SD, standard deviation; VPA, valproic acid.

Conversely, decreased total swimming distance, average swimming velocity, and clockwise rotation frequency, along with increased counter-clockwise rotation frequency, observed in the VPA+MOD group, could reveal hypolocomotion and impairments in decision-making behavior (Figure 2A–F). No major changes were observed in locomotory parameters, except for an increased counter-clockwise rotation frequency in healthy individuals exposed to MOD (Figure 2A–F). Decreased inactivity time and significantly decreased freezing behavior were also observed in the VPA+MOD group, as compared to the VPA+API group (VPA+MOD vs. VPA+API, *P* ═ 0.0321 in one-way ANOVA test) (Figure 2C, F).

Regarding anxiety-like behavior, our results showed mild anxiogenic effects in the VPA-treated group, described by increased thigmotaxis and time spent in the bottom area (Figure 3C, D). Furthermore, increased time spent in the top and crossing frequencies (bottom to top and peripheral to central) suggest impaired locomotory behavior (Figure 3A, B, E).

**Figure 3.**
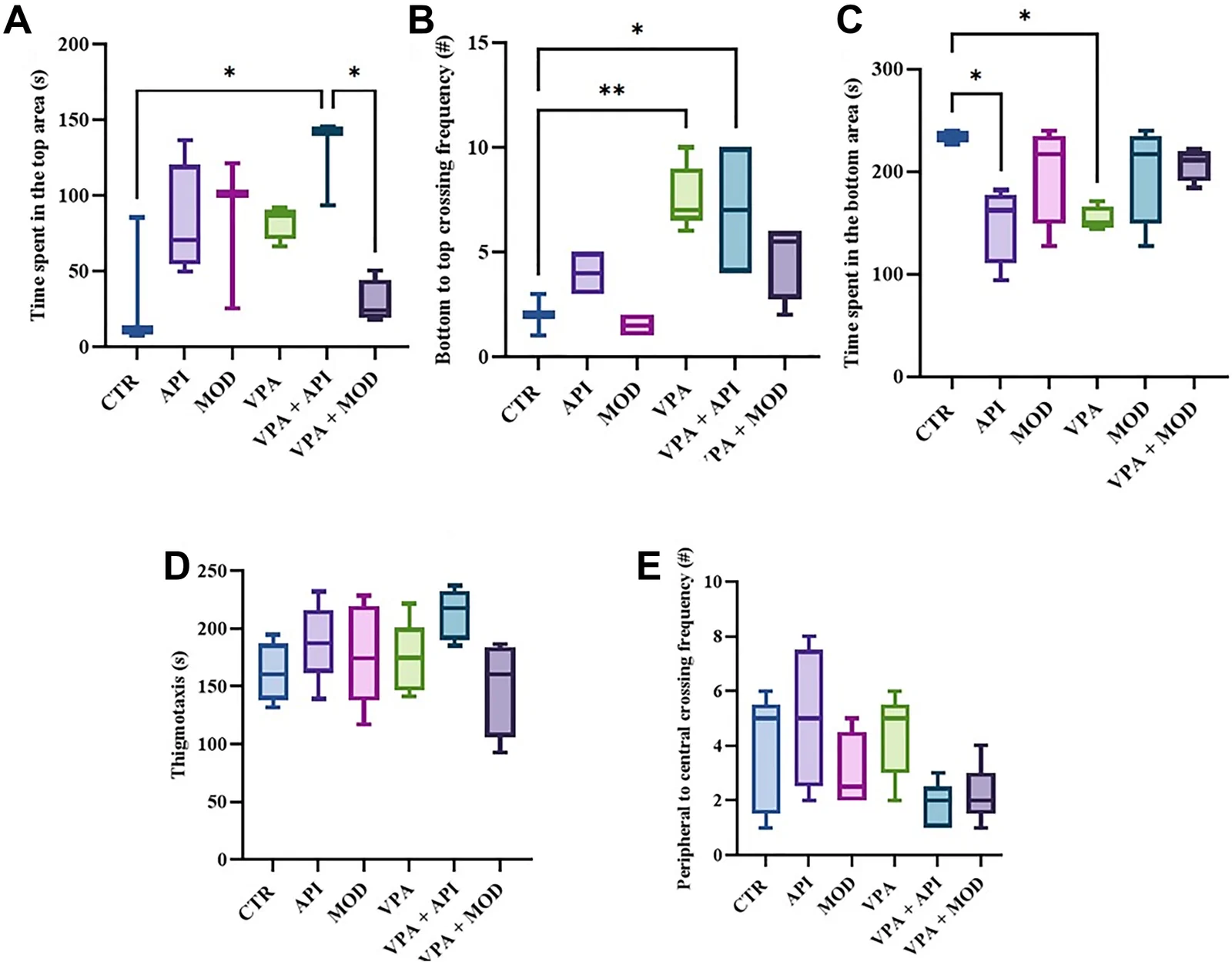
**Effects of API and MOD on anxiety-like behaviour in the novel tank test.** Anxiety-related behavioural responses were evaluated 24 h after the final treatment using the novel tank test. The analyzed parameters included: (A) time spent in the top area, (B) bottom-to-top crossing frequency, (C) time spent in the bottom area, (D) thigmotaxis, and (E) peripheral-to-central crossing frequency. VPA exposure was associated with altered vertical exploration and increased bottom-related behaviour, suggesting anxiety-like behavioural changes. API and MOD differentially modulated these responses in healthy and VPA-treated zebrafish, with significant changes observed in top-area exploration, bottom-to-top transitions, and time spent in the bottom area. Data are presented as box-and-whisker plots; boxes indicate the interquartile range, horizontal lines indicate the median, and whiskers indicate SD. Statistical significance was assessed using one-way ANOVA followed by post hoc analysis. *P < 0.05; **P < 0.01. **Abbreviations**: ANOVA, analysis of variance; API, first natural product based on honey; CTR, control; MOD, second natural product based on plant extracts; NTT, novel tank test; SD, standard deviation; VPA, valproic acid.

We observed a significant increase in time spent in the top area in the VPA+API group, as compared to the CTR group (VPA+API *vs.* CTR, *P ═* 0.0332 in one-way ANOVA; VPA+API vs. VPA+MOD, *P ═* 0.0137 in one-way ANOVA) (Figure 3A). The bottom-to-top crossing frequency also significantly increased in the VPA and VPA+API groups, as compared to CTR (VPA vs. CTR *P ═* 0.0054 in one-way ANOVA; VPA+API vs. CTR, *P ═* 0.0143 in one-way ANOVA) (Figure 3B). When administered to healthy individuals, API led to significantly decreased time spent in the bottom area (API vs. CTR, *P ═* 0.0254 in one-way ANOVA) and significantly increased bottom-to-top crossing frequency (API vs. CTR, *P ═* 0.0350 in one-way ANOVA) (Figure 3B, C). Similar outcomes, such as decreased time spent in the bottom area, decreased time spent in the top area, thigmotaxis, and variable crossing frequency, were registered in the VPA+MOD group (Figure 3A–E). Moreover, MOD administered to healthy individuals led to decreased time spent in both the top and bottom areas (Figure 3A, C).

### The effect of API and MOD on social behavior

Statistical analysis of locomotion behavior during the social behavior test revealed decreased total swimming distance, average swimming velocity, total inactivity time, and rotation frequencies in the VPA group, suggesting impairments in locomotory *behavior (e.g.*, total swimming distance, VPA vs. CTR, *P* ═ 0.0093 in one-way ANOVA test; *e.g.*, total inactivity time, VPA vs. CTR, *P <* 0.0001 in one-way ANOVA test) (Figure 4A–E). Similar behavioral outcomes, including significant decreases in total swimming distance and average swimming velocity, and significantly increased total inactivity time, were observed in both API and VPA+API groups, as compared to CTR and VPA *groups (e.g.*, average swimming velocity, API vs. CTR, *P* ═ 0.0092; VPA+API vs. CTR, *P ═* 0.0064 in one-way ANOVA test; *e.g.*, total inactivity time, API vs. CTR, *P <* 0.0011; VPA+API vs. CTR, *P ═* 0.0002; VPA+API vs. VPA, *P <* 0.0001 in one-way ANOVA test) (Figure 4A–C). Moreover, both MOD and VPA+MOD groups registered significantly decreased locomotion behavioral parameters, as compared to CTR and VPA groups *(e.g.*, total swimming distance, MOD vs. CTR, *P ═* 0.00093; VPA + MOD vs. CTR, *P* ═ 0.0006 in one-way ANOVA test; average swimming velocity, MOD vs. CTR, *P ═* 0.0050; VPA + MOD vs. CTR, *P ═* 0.0002 in one-way ANOVA test; total inactivity time, MOD vs. CTR, *P* ═ 0.0003; VPA+MOD vs. VPA, *P ═* 0.0043 in one-way ANOVA test) (Figure 4A–C). No major changes were noticed in rotation frequencies (Figure 4D, E).

**Figure 4.**
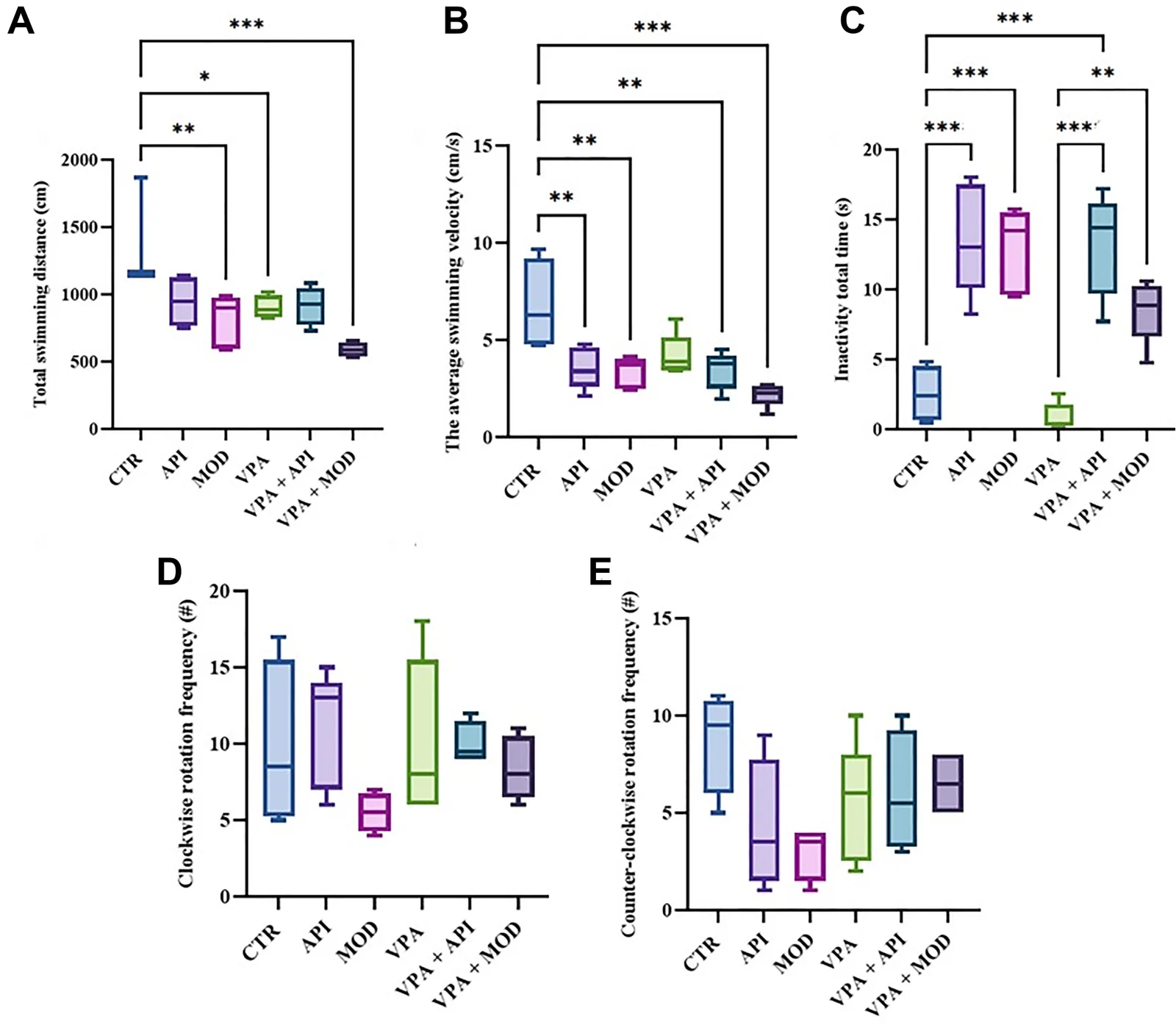
**Effects of API and MOD on locomotor performance during the social behaviour test.** General locomotor activity recorded during the social behaviour test was evaluated 24 h after the final treatment. The assessed parameters included: (A) total swimming distance, (B) average swimming velocity, (C) total inactivity time, (D) clockwise rotation frequency, and (E) counter-clockwise rotation frequency. VPA exposure was associated with impaired locomotor performance, while administration of API and MOD, either alone or following VPA exposure, further modified locomotor readouts. In particular, significant reductions in total swimming distance and average swimming velocity, together with significant changes in inactivity time, were observed in several treatment groups, whereas no major differences were detected in rotation frequencies. Data are presented as box-and-whisker plots; boxes represent the interquartile range, center lines indicate the median, and whiskers indicate SD. Statistical significance was determined using one-way ANOVA followed by post hoc analysis. *P < 0.05; **P < 0.01; ***P < 0.001. **Abbreviations**: ANOVA, analysis of variance; API, first natural product based on honey; CTR, control; MOD, second natural product based on plant extracts; SD, standard deviation; VPA, valproic acid.

Regarding social behavior assessments, our results showed no major changes after exposure to VPA, API, and MOD. In the VPA-exposed group, no significant changes were noticed in the time spent near social stimuli and the entry frequency into the stimuli arm (Figure 5A, B). However, the time spent in the right arm significantly decreased, as compared to the CTR group *(e.g.*, time spent in right arm, VPA vs. CTR, *P <* 0.0001 in one-way ANOVA test), while the time spent in the central arm and decision point increased (Figure 5A–D). Conversely, significantly increased time spent near social stimuli, decreased entry frequency into the stimuli arm, and significantly decreased time spent in other maze’s arms were recorded after API administration in both healthy and VPA-treated individuals, as compared to CTR and VPA groups *(e.g.*, time spent near social stimuli, API vs. CTR, *P ═* 0.0007; VPA+API vs. VPA, *P ═* 0.0445 in one-way ANOVA test; entry frequency into the stimuli arm, API vs. CTR, *P ═* 0.0184 in one-way ANOVA test; time spent in the right, API vs. CTR, *P <* 0.0001; VPA + API vs. CTR, *P <* 0.0001 in one-way ANOVA test; time spent in the central arm, API vs. CTR, *P ═* 0.0087; VPA+API vs. CTR, *P ═* 0.0062; VPA+API vs. VPA, *P ═* 0.030 in one-way ANOVA test) (Figure 5A–E).

**Figure 5.**
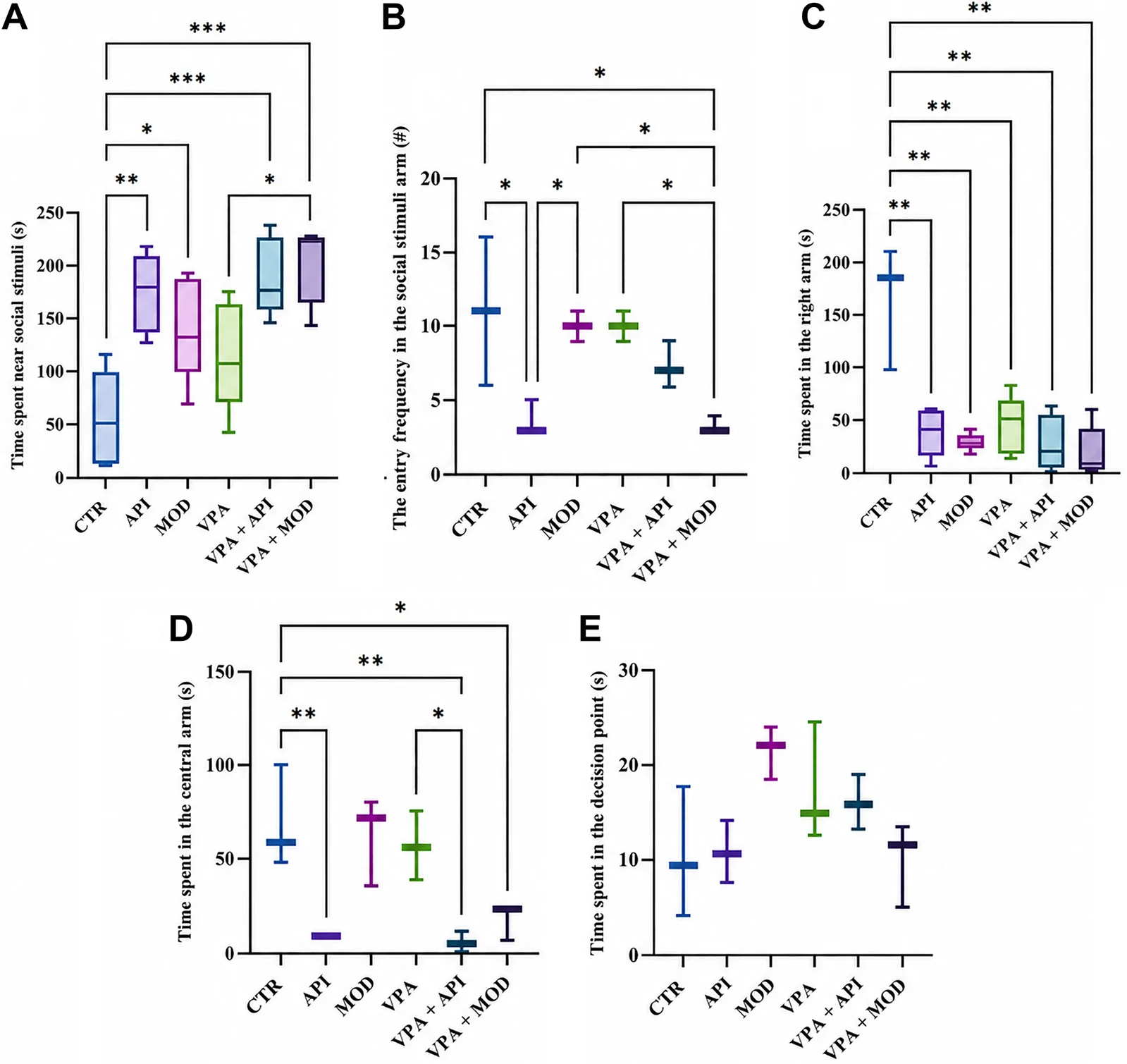
**Effects of API and MOD on social behaviour in the social behaviour test.** Social behaviour was assessed 24 h after the final treatment using a T-maze social behaviour test. The evaluated parameters included: (A) time spent near the social stimulus, (B) entry frequency into the social stimulus arm, (C) time spent in the right arm, (D) time spent in the central arm, and (E) time spent in the decision point. VPA exposure did not produce major changes in the primary social preference parameters, whereas administration of API and MOD, both in healthy and VPA-treated zebrafish, significantly modified social exploration. In particular, both natural products were associated with increased time spent near the social stimulus and reduced time spent in the non-social maze compartments, while changes in entry frequency into the stimulus arm were also observed. Data are presented as box-and-whisker plots; boxes represent the interquartile range, center lines indicate the median, and whiskers indicate SD. Statistical significance was determined using one-way ANOVA followed by post hoc analysis. *P < 0.05; **P < 0.01; ***P < 0.001. **Abbreviations**: ANOVA, analysis of variance; API, first natural product based on honey; CTR, control; MOD, second natural product based on plant extracts; SD, standard deviation; VPA, valproic acid.

On the other hand, similar behavioral outcomes were observed after MOD administration to healthy and VPA-treated individuals. This treatment significantly increased the time spent near social stimuli and significantly decreased entry frequency into the social stimuli arm, as well as time spent in other maze arms, compared to CTR and VPA groups *(e.g.*, time spent near social stimuli: MOD vs. CTR, *P ═* 0.0461; VPA+MOD vs. CTR, *P ═* 0.0002; VPA+MOD vs. VPA, *P ═* 0.0445 in one-way ANOVA; entry frequency in the social stimuli arm: VPA+MOD vs. CTR, *P ═* 0.0136; VPA+MOD vs. VPA, *P ═* 0.0338; VPA+MOD vs. MOD, *P ═* 0.0338 in one-way ANOVA; time spent in the right arm: MOD vs. CTR, *P <* 0.0001; VPA+MOD vs. VPA, *P <* 0.0001 in one-way ANOVA; time spent in the central arm: VPA+MOD vs. CTR, *P ═* 0.027 in one-way ANOVA) (Figure 5A–E).

### The Effect of API and MOD on Intestinal Microbiota Composition and Diversity

Intestinal microbiota shifts following VPA, API, and MOD treatments were investigated through 16S rRNA gene profiling. Nanopore sequencing of intestinal microbiome samples generated a total of 24,790 reads, corresponding to an estimated yield of 52.38 Mb. Read length distribution exhibited a median N50 value of 1.73 kb, consistent with near full-length 16S rRNA gene amplicons. Raw genus-level count data were assigned to corresponding barcoded intestinal samples and utilized for downstream microbiota analyses. Although a 24-barcode library was employed, only barcodes 01–18 corresponded to biological samples included in the experimental design, representing three randomly selected individuals from each of the six groups. Genus-level read depths varied substantially among these samples, ranging from 1–3084 reads per barcode. To mitigate bias from low-yield samples, a minimum inclusion threshold of ≥10 genus-level classified reads was applied. The proportion of unclassified or unmapped reads remained low across samples, generally below 2%, suggesting high base-calling accuracy and efficient reference-based classification. Following quality filtering and taxonomic assignment using the wf-16s pipeline, most reads were retained and classified at least to the phylum level, indicating that sequencing depth and overall data quality were adequate for robust characterization of intestinal microbial communities.

**Figure 6.**
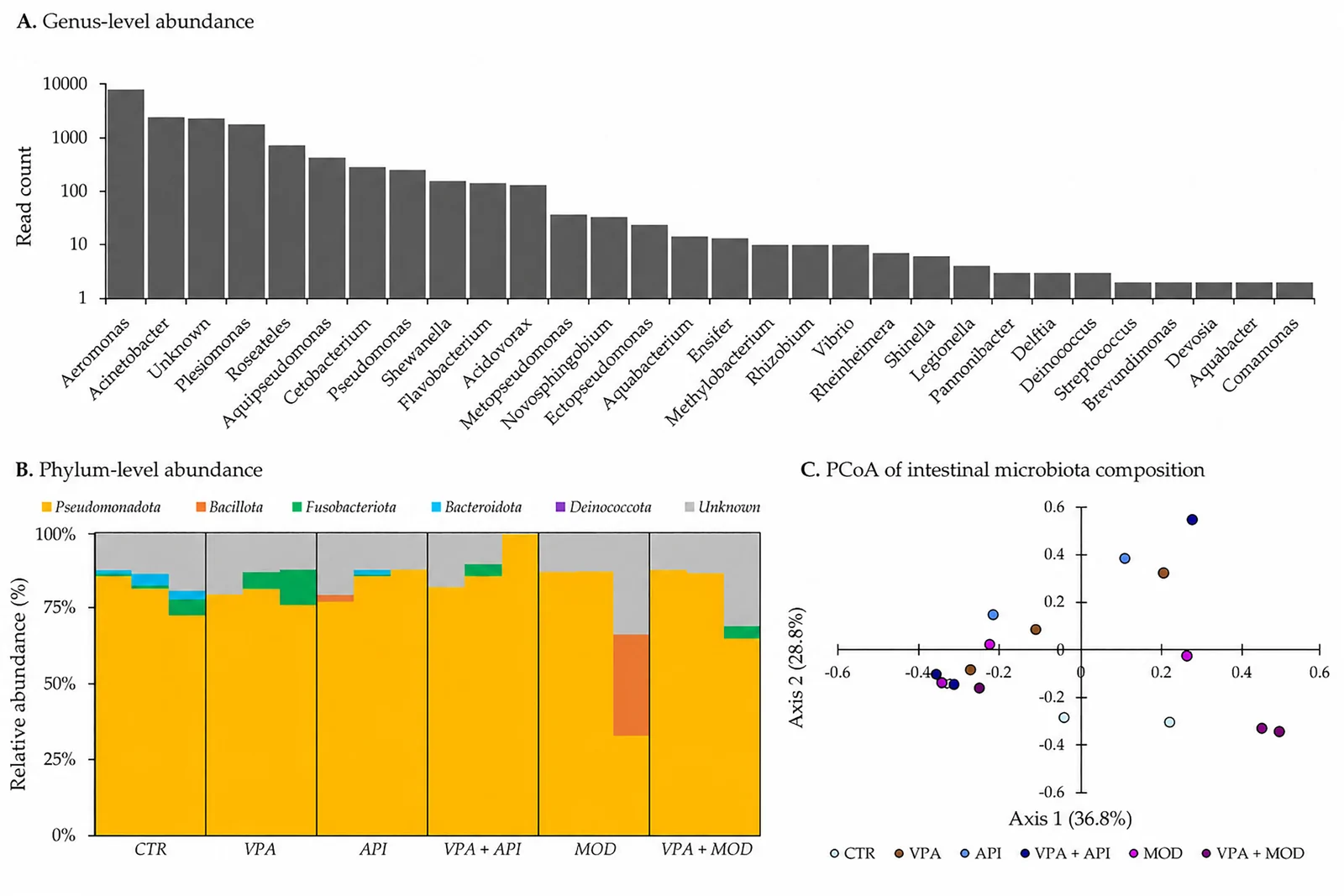
**Intestinal microbiota composition and community structure across experimental groups.** Intestinal microbiota profiling was performed using 16S rRNA gene sequencing. (A) Genus-level read counts for the dominant bacterial genera detected across intestinal samples. Read counts are displayed on a logarithmic scale. Aeromonas, Acinetobacter, Plesiomonas, and unclassified taxa were the most abundant components of the microbial community. (B) Phylum-level relative abundance across experimental groups, expressed as percentages and displayed as stacked bars summing to 100%. Pseudomonadota predominated in all groups, whereas Bacillota, Fusobacteriota, Bacteroidota, Deinococcota, and unclassified taxa were detected at lower relative abundances. API and MOD treatments, particularly when combined with VPA, were associated with descriptive shifts in secondary phyla and unclassified taxa. (C) Principal coordinates analysis based on Bray-Curtis dissimilarity at the genus level. Each point represents one intestinal sample and is colored according to the experimental group. Axes 1 and 2 explained 36.8% and 28.8% of the total variance, respectively. The ordination showed partial clustering with substantial overlap among groups, and PERMANOVA did not indicate statistically significant group separation; therefore, the microbiota patterns should be interpreted as exploratory descriptive trends. **Abbreviations:** API, first natural product based on honey; CTR, control; MOD, second natural product based on plant extracts; PCoA, principal coordinates analysis; PERMANOVA, permutational multivariate analysis of variance; rRNA, ribosomal RNA; VPA, valproic acid.

At the phylum level, the intestinal microbiota was overwhelmingly dominated by *Pseudomonadota*, accounting for 83.84% of all classified reads across samples. Minor bacterial phyla included *Fusobacteriota* (1.64%) and *Bacteroidota* (0.83%), whereas *Bacillota* and *Deinococcota* were detected at very low relative abundances (<0.05% each). At the genus level, the microbial community was largely composed of taxa belonging to the phylum *Pseudomonadota*, with *Aeromonas* (48.03% of total reads), *Acinetobacter* (14.37%), and *Plesiomonas* (10.50%) representing the most abundant genera (Figure 6A). Additional genera, including *Roseateles*, *Aquipseudomonas*, *Cetobacterium*, *Pseudomonas*, *Shewanella*, and *Flavobacterium*, were detected at lower relative abundances, while 13.65% of reads remained unclassified at both phylum and genus levels. Overall, the intestinal microbiota was characterized by a limited number of highly dominant taxa, with the remaining genera contributing only marginally to community composition. Across experimental groups, all communities remained predominantly enriched in *Pseudomonadota*, suggesting a relatively conserved core microbiota structure (Figure 6B).

**Table 1:** Summary of alpha diversity indices across experimental groups

**Experimental group**	**Berger Parker index**	**Effective number of species**	**Inverse Simpson’s index**	**Pielou’s evenness**	**Richness**	**Shannon diversity index**	**Simpson’s index**
CTR	0.55	3.60	1.76	0.59	10.33	1.26	0.60
API	0.54	4.45	1.78	0.58	11.67	1.38	0.62
MOD	0.56	3.24	2.17	0.70	5.67	1.09	0.55
VPA	0.37	5.32	1.35	0.85	7.67	1.66	0.75
VPA + API	0.64	2.40	2.40	0.59	5.67	0.86	0.46
VPA+MOD	0.77	1.94	3.27	0.49	4.33	0.63	0.35

Values represent group means calculated from rarefied, genus-level data after applying a minimum read-depth threshold (≥10 reads). The number of samples retained per group after filtering was *n* ═ 3 for all groups. **Abbreviations**: API, first natural product based on honey; CTR, control; MOD, second natural product based on plant extracts; VPA, valproic acid.

The VPA-treated group showed increased proportions of *Fusobacteriota* and unclassified taxa compared to the CTR group, suggesting mild treatment-related alterations in microbial composition. The API-treated group remained broadly comparable to CTR, whereas administration of API to VPA-treated zebrafish resulted in an almost exclusive dominance of *Pseudomonadota*, suggesting a simplified taxonomic profile. Conversely, the MOD-treated group exhibited the most pronounced shift in phylum-level composition, with an increase in *Bacillota* abundance accompanied by a decrease in *Pseudomonadota*. Furthermore, the VPA + MOD group preserved the dominance of *Pseudomonadota* but also displayed an increased proportion of unclassified taxa, indicating persistent compositional changes under combined treatment. Collectively, these results suggest that although *Pseudomonadota* remained dominant in all groups, both API and MOD administration, particularly in combination with VPA, induced alterations in the abundance of secondary phyla and unclassified taxa. Due to the exploratory design and variable sequencing depth, taxonomic patterns are presented as descriptive trends rather than definitive treatment-induced differences.

Alpha diversity indices, calculated at the genus level after rarefaction to 100 reads (corresponding to the lowest read depth among retained samples) using 1000 rarefaction iterations, revealed differences in microbial richness, dominance, and evenness across treatment groups (Table 1). Descriptively, the VPA group exhibited higher mean richness and Shannon diversity than CTR, whereas VPA+API and VPA+MOD showed lower mean richness and Shannon diversity and higher Berger-Parker dominance. However, Kruskal-Wallis testing did not detect statistically significant differences among groups for any alpha-diversity index after FDR correction: richness (*H* ═ 8.4167, *P ═* 0.1347, FDR-adjusted *P ═* 0.2536), Shannon diversity (*H* ═ 6.9833, *P ═* 0.2219, FDR-adjusted *P ═* 0.2536), Simpson index 1-D (*H* ═ 7.8167, *P ═* 0.1666, FDR-adjusted *P ═* 0.2536), inverse Simpson index 1/D (*H* ═ 7.2083, *P ═* 0.2056, FDR-adjusted *P ═* 0.2536), Pielou’s evenness (*H* ═ 6.4167, *P ═* 0.2678, FDR-adjusted *P ═* 0.2678), Berger-Parker index (*H* ═ 7.7500, *P ═* 0.1706, FDR-adjusted *P ═* 0.2536), effective number of species (*H* ═ 7.3750, *P ═* 0.1942, FDR-adjusted *P ═* 0.2536), and Fisher’s alpha (*H* ═ 8.4167, *P ═* 0.1347, FDR-adjusted *P ═* 0.2536).

The VPA-treated group exhibited the lowest Berger–Parker index along with the highest effective number of species, Shannon diversity, Simpson’s index, and Pielou’s evenness, suggesting a more evenly distributed microbial community. Similarly, the API-treated group showed the highest richness and a relatively complex microbial structure compared to the CTR group. In contrast, the VPA+API group displayed increased dominance and lower richness and Shannon diversity, suggesting reduced microbial complexity following combined treatment. The MOD-treated group showed intermediate alpha diversity values, suggesting a less complex but still relatively balanced microbial community. Interestingly, the VPA+MOD group exhibited the highest Berger–Parker index together with the lowest effective number of species, richness, Shannon diversity, and Pielou’s evenness, indicating a microbial structure strongly dominated by a limited number of taxa.

Community clustering, assessed by Principal Coordinates Analysis (PCoA) based on Bray–Curtis dissimilarity, showed that the first two axes explained 65.6% of the total variance in microbial community composition (Figure 6C). Samples exhibited only partial grouping according to experimental conditions, with substantial overlap among groups. The VPA and VPA+API groups appeared more dispersed in the ordination space, suggesting increased intra-group variability, whereas the MOD group showed a tighter distribution. Notably, the VPA+MOD group formed the clearest cluster, suggesting a more distinct and homogeneous microbial community configuration under combined treatment. However, PERMANOVA did not indicate significant group separation among experimental groups (pseudo-F = 1.3335, R2 = 0.4256, *P ═* 0.2548, 9999 permutations). Differential abundance testing at the genus level also did not identify taxa that remained statistically significant after Benjamini-Hochberg FDR correction. The lowest unadjusted *P*-value was observed for *Methylobacterium* (*P ═* 0.0407), but this did not remain significant after FDR adjustment (FDR-adjusted *P ═* 0.4320). Therefore, the microbiota data do not support statistically confirmed treatment-induced changes in alpha diversity, beta diversity, or genus-level differential abundance. Overall, these findings could indicate that API and MOD administration influenced both the taxonomic composition and diversity structure of the intestinal microbiota, with the most evident changes observed in the combined treatment groups, particularly VPA+MOD. Furthermore, the observed patterns should be interpreted as exploratory descriptive trends requiring validation in larger studies with more balanced sequencing depth.

## Discussion

The present study aimed to evaluate and describe the potential of two novel natural products—API and plant extract-based (MOD)—to modulate exploratory and socio-affective behavior in a VPA-based adult zebrafish model of ASD. Our VPA-based ASD model is characterized by mild changes in socio-affective behavior and altered locomotory behavior. These effects were further modulated by API when administered after VPA treatment, encompassing changes in both locomotory and social behaviors. Similarly, MOD treatment led to modifications of social and decision-making-associated behaviors.

Our model, based on a 5-day treatment with 300 µM VPA, was characterized by impaired exploratory *behavior (e.g.*, increased total swimming distance, decreased average swimming velocity, and changes in clockwise and counter-clockwise rotation frequency) and anxiety-like states *(e.g.*, increased freezing behavior, crossing frequency, thigmotaxis, and time spent in the top and bottom areas). As previously described by Kalueff et al. [23] in a comprehensive systematic review, anxiety-like behavior in zebrafish can be assessed using the NTT, including reduced exploration of the upper zone, increased bottom presence, erratic movements, and freezing. Furthermore, the sensitivity of the NTT as an anxiety paradigm is supported by Fontana et al. [33], who demonstrated a strong correlation between NTT performance and other assessments of anxiety-like behavior, confirming its validity as an independent measure in zebrafish. Moreover, no major changes were noticed in social behavior after VPA treatment (e.g., time spent near social stimuli and entry frequency into the stimuli arm). However, impaired exploratory behavior coupled with anxiety-like states may contribute to an apparent arm preference, as shown by increased time spent in other maze arms (e.g., central arm and decision point).

In similar experimental conditions (300 µM VPA treatment for 5 days), Chele et al. [37] reported minor impairments of locomotor performance *(e.g.*, decreased total swimming distance and average velocity), while Blazina et al. [38] also reported locomotor performance impairments after even shorter VPA treatments (265 µM VPA for 10 min). Regarding social behavior, Chele et al. reported that VPA treatment did not induce major changes and fish tended to spend more time near social stimuli. Moreover, Dreosti et al. [39] and Liu et al. [40] reported that VPA-treated zebrafish did not exhibit overt social behavior, while Blazina et al. [38] found that zebrafish larvae exposed to VPA showed no changes in social behavior and tended to spend more time in the distal area and less time near the stimulus at the adult stage. Conversely, a previous study has explained that the paradoxical increase in social behavior could be the result of moderate VPA administration and age [41], thus highlighting the time- and dose-dependent behavioral effect of VPA on social interaction [42–45].

When API was administered to VPA-treated individuals, impairments in exploratory behavior were observed, characterized by increased total swimming distance, average swimming velocity, and inactivity time. Additionally, rotation frequencies were altered, with decreased clockwise rotation and increased counter-clockwise rotation. Elevated anxiety-like states, evidenced by significantly increased freezing behavior, were also noted. These findings align with previous research by Paduraru et al. [14], who reported significantly increased inactivity time following similar API treatment (500 mg/L).

Regarding social behavior, both our findings and prior studies indicate that API significantly modifies the time spent near social stimuli in healthy individuals [14]. Furthermore, while another honey product, bee pollen, did not modulate socio-affective behaviors in healthy individuals, it was shown to alter social behavior and increase anxiety states *(e.g.*, significantly increased freezing time) in VPA-treated individuals [46]. Conversely, Nigerian honey, another honey product, exhibited anxiolytic effects but impaired locomotion, attributed to central inhibitory effects on glutamatergic, dopaminergic, and GABAergic systems [47]. Similarly, Gavrilović et al. [48] proposed that honey products could positively modulate serotonergic systems in socially isolated animals, thereby maintaining peripheral serotonergic system homeostasis under stress conditions [49].

In VPA-treated zebrafish, moringa oleifera extract (MOD) treatment resulted in hypolocomotion and impaired decision-making behavior, manifested as decreased total swimming distance, average swimming velocity, and clockwise rotation frequency, alongside increased counter-clockwise rotation frequency. While some anxiety parameters suggested mild anxiolytic effects *(e.g.*, significantly decreased freezing behavior, thigmotaxis, and total inactivity time), a decrease in time spent in the top area was also observed. MOD also significantly altered the time spent near social stimuli in both healthy and VPA-treated zebrafish. However, it is crucial to consider that hypolocomotion may influence specific socio-affective behavioral parameters, potentially mimicking an ”enhancement” for particular stimuli or T-maze compartments by limiting exploratory behavior rather than genuinely reflecting social interest [39, 50].

Previous research has demonstrated that plant extracts and vegetable oils can mitigate ASD-specific behaviors by modulating oxidative stress, which contributes to brain inflammation and subsequent behavioral alterations [51]. In this context, various vitamins and antioxidant compounds, including carotenoids, polyphenols, and flavonoids, have been reported to modulate affective impairments and sensorimotor responses [51–53]. Beyond traditional zebrafish models, complex transgenic lines such as Tg(kdrl:mCherry) and Tg(fli1:green fluorescent protein [GFP]) offer potential for personalized therapy [17, 18]. Evaluating the translational potential of these experimental models includes novel methods for *in vivo* clinical implementation, such as Patient-Derived Xenograft (PDX) models [17, 18].

The analysis of the intestinal microbiota in this study should be considered exploratory. Following sequencing, application of a minimum read-depth threshold, rarefaction for alpha diversity, and relative abundance normalization for beta diversity and differential abundance testing, no statistically significant differences were detected in alpha diversity. Furthermore, PERMANOVA revealed no significant group separation, and no genus remained significant after FDR correction. Consequently, the data do not statistically support the observed tendency for API and MOD to promote microbial diversity. Instead, the results suggest descriptive, context-dependent trends in microbiota composition that necessitate confirmation in larger, adequately powered studies with more balanced sequencing depth.

Descriptively, the intestinal microbiota across all experimental groups remained predominantly composed of *Pseudomonadota*, with *Fusobacteriota*, *Bacteroidota*, *Bacillota*, and *Deinococcota* detected at lower relative abundances [16]. API-treated healthy fish exhibited a relative increase in *Pseudomonadota*, while *Bacteroidota* and *Deinococcota* maintained very low relative abundances. MOD-treated healthy fish displayed a generally similar profile, with minor differences noted in *Bacteroidota* abundance. In VPA-treated groups, neither API nor MOD restored microbiota diversity, and microbial profiles remained largely comparable to the VPA group, characterized by high relative abundance of *Pseudomonadota* and low relative abundance of *Fusobacteriota*. Therefore, these patterns should be interpreted as descriptive trends rather than statistically confirmed treatment effects.

These exploratory observations align with previous studies reporting altered gut microbiota composition in ASD-related models, including enrichment of *Pseudomonadota* and reduced *Bacteroidota* following VPA exposure [54]. Moreover, imbalances such as increased *Pseudomonadota* and decreased *Bacillota* in the gut microbiota of ASD individuals have been hypothesized to affect tryptophan metabolism, potentially reducing serotonin synthesis and influencing social behavior [55–57]. Prior research also indicates that honey products may modulate intestinal microbiota, with increased *Lactobacilli* and *Bifidobacteria* observed after two months of treatment [48]. Similarly, rodent studies have shown that honey treatment can influence chronic ethanol-induced gastric injury and microbial composition, while prolonged administration of polyphenol-rich plant extracts or essential-oil-rich preparations may modulate intestinal microbiota diversity [58–61].

Several limitations of the current study warrant consideration. The small sample size (*n* ═ 5 per group for behavioral testing and *n* ═ 3 per group initially selected for exploratory microbiota profiling) may have limited statistical power and detection sensitivity. For microbiota analyses, read depth varied substantially among samples, and three low-yield samples were excluded based on a predefined threshold of ≥ 10 reads, resulting in an uneven sample distribution across groups. The absence of extraction blanks or sequencing negative controls means that low-level reagent or environmental contamination cannot be entirely ruled out. Furthermore, each experimental group was housed in a single tank without replication, precluding the complete exclusion of tank-specific effects and suggesting that behavioral responses may have been partially influenced by shared environmental conditions within tanks.

Secondly, the short treatment duration and the dosages of natural products (API, MOD) employed may have constrained their ability to modulate target behaviors and intestinal microbiota, as suggested by recent studies [58–61]. Consequently, interpretations regarding biological mechanisms should remain cautious. Additionally, the administration of honey and plant-extract syrup in an aquatic zebrafish model could introduce confounding effects related to water quality and physicochemical parameters. Despite meticulous efforts to prepare fresh solutions and maintain regular water changes, these factors cannot be entirely excluded and should be considered when interpreting behavioral or microbiome changes. A significant limitation is that direct administration of honey and plant-extract syrup into the water could alter pH, dissolved oxygen, conductivity, ammonia/nitrite levels, turbidity, or other water quality parameters. Future studies should rigorously monitor water quality parameters to ensure that observed behavioral or microbiome changes are not erroneously attributed to extract activity rather than natural responses to water quality fluctuations.

Regarding social behavior assessments, the systematic review by Ogi et al. [31] highlighted that social preference in zebrafish can be evaluated through numerous behavioral assays. However, aspects of experimental design, such as the number of social stimuli, can be debated when assessing multiple specific social traits. In the current design, a single social stimulus with two non-social areas was utilized, focusing on social approach rather than inter-stimulus preference. Furthermore, Ogi et al. [31] recommend an interaction phase of up to 6 min, during which exploratory behavior increases and behavioral inhibition decreases, allowing for a more reliable assessment of sociability. The present protocol employed a 30-second acclimatization phase and a 4-minute test phase, which may have limited the expression of assessed behaviors and should be considered when interpreting social behavior results. Therefore, these findings should be interpreted with caution as exploratory and descriptive.

## Conclusion

Natural products derived from honey and plant extracts have a long history in traditional medicine, valued for their neuroprotective and antibacterial properties. Enriching honey with plant-derived additives, including extracts and essential oils, can enhance its content of bioactive compounds. Our results indicate that both natural products are associated with changes in exploratory and socio-affective behavior in both healthy and VPA-treated zebrafish. Furthermore, intestinal microbiota analysis revealed descriptive, treatment-associated trends in microbial composition, demonstrating that VPA can limit the number of highly dominant genera. While both natural products were associated with slight variations in microbial diversity, these findings should be interpreted cautiously and require further validation in larger-scale studies.

## Data Availability

Data is contained within the article.
